# Distribution of toxin genes among different *spa* types and phage types of animal *Staphylococcus aureus*

**DOI:** 10.1007/s00203-015-1127-y

**Published:** 2015-06-25

**Authors:** Katarzyna Garbacz, Lidia Piechowicz, Aneta Mroczkowska

**Affiliations:** Department of Medical Microbiology, Medical University of Gdansk, 38 Do Studzienki St., 80-227 Gdańsk, Poland; Department of Epidemiology and Clinical Microbiology, National Medicines Institute, 30/34 Chełmska St., 00-725 Warsaw, Poland

**Keywords:** Animal *S. aureus*, *spa* typing, Phage typing, Toxin genes

## Abstract

We analyzed distribution of toxin genes (*sea*-*seo*, *eta*, *etb*, *tst*, *lukS/lukF*-*PV*) among *spa* types and phage types of 39 *Staphylococcus aureus* (*S. aureus*) isolates from healthy and diseased animals. All isolates turned out to be *mecA* negative (MSSA). Nine *spa* types were identified: t144 and t723 (dogs), t084 (dogs and pigs), t5447 (cat), t1491 and t008 (pigs), t002, t127 and t3478 (poultry). Seven phage types were detected, enclosed within four phage groups: I (cat), II (dogs), III (pigs) and mixed group (dogs and pigs). Three poultry *spa* types proved to be non-typeable by phages. Toxin genes were detected in 33 out of the 39 animal isolates. Our analysis revealed that the incidence of some toxin genes in *S. aureus* is host specific. Canine isolates t144 of phage group II harbored exfoliative toxin gene (*eta*), and porcine isolates type t1491 representing phage group III showed enterotoxin A gene (*sea).* The enterotoxin gene cluster (*egc1)* and enterotoxin gene *seh* were found in non-typeable isolates from chicken and in one feline isolate type t5447.

## Introduction

Although *Staphylococcus aureus* is mostly a widely spread human pathogen, it can be also isolated from animals (Devriese 1984; Sung et al. 2008). The distribution of specific *S. aureus* strains among hosts of various species is an interesting albeit still poorly understood issue. Although staphylococcal strains vary depending on the host, some types can be isolated from both humans and animals. This was confirmed by recent molecular studies, as well as by previous research based on phage typing (Hasman et al. 2010; Krynski et al. 1981). Phage typing was one of the first methods used to analyze the relatedness of staphylococcal strains. However, nowadays, this method was nearly entirely replaced by genotyping, mostly due to increasing prevalence of strains that are non-typeable by phages. Nevertheless, phage typing can provide valuable information on the prevalence and evolution of certain human and animal *S. aureus* isolates (Krynski et al. 1981; Vintov and Aarestrup 2003; Vintov et al. 2003). Nowadays, *spa* typing is the most widely used method for the first-line typing and epidemiological analyses. This method determines the sequence variation of the polymorphic region *X* of the *spa* gene for *S. aureus* surface protein A. The diversity of the *spa* gene, consisting mainly of a number of repeats of 24 bp in length, is attributed to point mutations, as well as to deletions and duplications of the repeats. The *spa* typing allows us to characterize and compare strains isolated from various hosts for their phylogenetic relatedness (Ebner et al. 2013; Espinosa-Gongora et al. 2012; Vincze et al. 2013). *S. aureus* is known to synthesize an array of virulence factors, such as leukocidins, exfoliative toxins and pyrogenic toxins superantigens (PTSAgs). These toxins cause a variety of diseases in humans (Bukowski et al. 2010; Krakauer 2013). *S. aureus* strains colonizing various animals and humans may differ in terms of generated toxins (Gomez-Sanz et al. 2013). Synthesis of toxins by animal *S. aureus* isolates constitutes another important and poorly understood issue.

The aim of this study was to determine distribution of toxin genes among *spa* types and phage types of animal isolates of *S. aureus*.

## Results

A total of 39 *S. aureus* isolates were identified, among them 16 canine (6 nasal isolates from healthy dogs and 10 isolates from skin lesions), one feline isolate from a cat with conjunctivitis, 15 porcine isolates from pigs with skin lesions and 7 poultry isolates from chicken with arthritis (*n* = 6) and septicemia (*n* = 1).

Genotyping of isolated *S. aureus* revealed nine *spa* types. Three *spa* types (t144, t084 and t723) of canine isolates were identified. The most prevalent of them was t144, found in 8 out of the 16 isolates. In turn, t1491 (*n* = 12) turned out to be the most prevalent among 15 porcine isolates, followed by t008 and t084, isolated from two and one animals, respectively. Poultry *S. aureus* isolates were represented by three types: t002 (*n* = 3), t127 and t3478 (*n* = 2 each). The only feline isolate represented type t5447, not observed among any other animal strains (Table 1).

**Table 1:** Distribution of *spa* types, phage types and toxin genes in animal *S. aureus*

Host species	Number of isolates (c/i)	*Spa* type	*Spa* repeats	*Predicated ST* ^*a*^	Phage type	Phage group	Toxin gene profile
Dog	8 (6/2)	t144	07-23-12-34-34-12-12-23-02-02-12-23	ST15 or ST18	3C/55/71	II	*eta*
Dog	5 (0/5)	t723	11-19-12-34-22-25	ST8	29/52/52A/79/80/81/6/42E/47/53/54/75/83A/84/85/88/89	I + III	*sea*
Dog	3 (0/3)	t084	07-23-12-34-34-12-12-23-02-12-23	ST15 or ST18	89/29/52/52A/84/85/88/89	I + III	none
Cat	1 (0/1)	t5447	07-02-16-02-25-17-24	–	29/79/81	I	*egc1* ^*b*^
Pig	12 (0/12)	t1491	07-23-21-17-13-34-34-16-34-33-13	ST1	75/84/85	III	*sea*
Pig	2 (0/2)	t008	11-19-12-21-17-34-24-34-22-25	ST8	6/42/53/54	III	none
Pig	1 (0/1)	t084	07-23-12-34-34-12-12-23-02-12-23	ST15 or ST18	52A/53/75/83A/84/85	I + III	none
Chicken	3 (0/3)	t002	26-23-17-34-17-20-17-12-17-16	ST5	NT	NT	*egc1* ^*b*^
Chicken	2 (0/2)	t127	07-23-21-16-34-33-13	ST1	NT	NT	*sec, seh*
Chicken	2 (0/2)	t3478	26-23-17-34-17-20-17-12-17-16-12-17-16	ST5	NT	NT	*egc1* ^*b*^

All isolates, irrespective of their host, turned out to be *mecA* negative (MSSA)

*c/i* colonization/infection, *NT n*on-typeable

^a^ST type retrieved from the Ridom SpaServer

^b^ *egc1(seg, sei, sem, sen and seo)*

Phage typing revealed seven phage types representing four phage groups. The analyzed isolates belonged to three distinct phage groups (I–III), as well as to one mixed group (I + III). Phage group I was represented solely by one feline isolate (29/79/81 lytic pattern), not detected in any other host. Only canine isolates were typeable by group II phages (3C/55/71 lytic pattern). Most porcine isolates belonged to phage group III. The mixed group included canine and porcine isolates. Three poultry *spa* types proved to be non-typeable by phages (Table 1).

Toxin genes were detected in as many as 33 out of the 39 animal isolates. A total of 25 *S. aureus* isolates were found to be enterotoxigenic. The *sea* gene, found in 12 t1491 porcine isolates and 5 t723 canine isolates, proved to be the most frequent of classical PTSAg genes. The second most frequent PTSAg gene was *sec*, found in two chicken isolates representing t127 genotype. The *eta* gene was detected in eight canine isolates representing *spa* type t144. Five chicken-derived *S. aureus* isolates harbored the *egc1* gene grouping, comprising enterotoxin genes *seg*, *sei*, *sem*, *sen* and *seo*. Moreover, the *egc1* was carried the only one feline staphylococcal isolate. The *seh* gene was found solely in two t127 chicken isolates. The toxin genes were not harbored by any of t084 and t008 isolates. Moreover, we did not find *tst* and *lukS/F*-*PV* genes in our material.

## Discussion

We analyzed distribution of toxin genes among *spa* types and phage types of animal *S. aureus* from various hosts. Better understanding of diversity of toxigenic *S. aureus* circulating in an animal reservoir adds to current knowledge on pathogenicity of *S. aureus* species.

We identified three *spa* types (t144, t084 and t723) among canine staphylococcal isolates. On the basis of predicted ST15, one may assume that the t144 and t084 isolates likely belonged to CC15 clonal complex. This complex was considered a typical human-associated lineage thus far (Sung et al. 2008). As recent studies proved that it can be presented among both human and canine isolates, a transmission of strains from humans to dogs was likely to occur (Vincze et al. 2013). The same refers to another type isolated in our study, t723, which was originally identified as a human-associated strain from a subtropical recreational marine beach (Plano et al. 2013). This points to a possible transmission of *S. aureus* isolates from humans to dogs, especially that canine isolates were typeable by group II phages. To this date, strains of phage group II were generally isolated from skin lesions in humans (Rosdahl et al. 1990; Ladhani and Joannou 2000; Piechowicz et al. 2012). They are characterized by the ability to induce skin infections, such as impetigo and staphylococcal scalded skin syndrome, associated with the synthesis of exfoliative toxins types A and B (Ladhani and Joannou 2000; Kapral and Miller 1971). Canine isolates of phage group II were the only animal staphylococci identified in our study that harbored exfoliative toxin type A (*eta*) gene. Previous studies showed an association between synthesis of exfoliative toxins and susceptibility to phages of group II (Parker et al. 1955; Parker and Williams 1961). This relationship, previously reported solely in the case of human *S. aureus*, was also documented among the canine isolates analyzed in our study, which may point to their potential human origin. This hypothesis is further supported by the fact that the results of recent studies suggest that incidence of *S. aureus* among dogs is associated with their contact with humans (Gomez-Sanz et al. 2013).

The only feline isolate analyzed in our study represented *spa* type t5447, the sequence of which closely resembled that of t3001, likely belonging to CC45 complex (http://spaserver.ridom.de). To this date, type 5447 *S. aureus* was isolated from humans in Danish hospital (http://spaserver.ridom.de). Moreover, our t5447 feline isolate showed a unique lytic pattern (29/79/81) corresponding to phage group I, not detected in the case of strains from any other host. The feline isolate lacked classical PTSAg genes, but contained the enterotoxin gene cluster (*egc1*). The *egc* genes were previously detected in *S. aureus* strains from poultry and rabbits (Bystron et al. 2010; Vancraeynest et al. 2006). Moreover, this locus was present in a number of strains isolated from healthy humans, as well as from patients with septic shock and neonates with diarrhea (Naik et al. 2008; van Belkum et al. 2006). To the best of our knowledge, our isolate is the first reported t5447 feline strain of *S. aureus*.

t1491 turned out to be the predominant *spa* type among porcine isolates. It was previously isolated from the cases of porcine infection in Denmark (Hasman et al. 2010). The two remaining *spa* types, t008 and t084, were previously detected in humans, dogs and cattle (Hasman et al. 2010; Vincze et al. 2013). Phage typing showed that both t1491 and t008, characterized by 75/84/85 and 6/42/53/54 lytic patterns, respectively, belonged to phage group III. In turn, the t084 isolate represented mixed phage group (I + III). The presence of mixed phage groups among animal *S. aureus* isolates was reported previously (Vintov and Aarestrup 2003; Vintov et al. 2003). t1491 turned out to be the only isolate that harbored the *sea* toxin gene; the remaining two isolates were non-toxigenic. SEA is one of the first described PTSAgs (Betley and Mekalanos 1985). It constitutes the most important cause of staphylococcal food poisonings and is involved in invasive staphylococcal infections of humans (Pinchuk et al. 2010; Tristan et al. 2007). Therefore, the presence of SEA-positive isolates is associated with potential risk of food poisoning.

The presence of identified by us *spa* types among poultry isolates belonging to ST1 and ST5 was reported previously in various countries (Hasman et al. 2010; Ebner et al. 2013; Bystron et al. 2010; Monecke et al. 2013; Krupa et al. 2014). Recently, published Polish study revealed that t002 and t3478 were frequently isolated from the both chickens and chicken meat, indicating potential of introduction of animal-associated genotypes into food chain (Krupa et al. 2014). The staphylococci isolated from poultry were non-typeable by phages. This was likely associated with unique phenotypic and genotypic characteristics of poultry strains, distinguishing them unambiguously from their human and animal counterparts (Lowder and Fitzgerald 2010). Recently, several mobile novel elements, not found in human or animal isolates but widely distributed among poultry *S. aureus* strains, were detected (Lowder and Guinane 2009). Isolates representing ST5 showed the presence of *egc1*, but lacked genes for other toxins. Nevertheless, *egc* locus was not universally harbored by all the poultry strains, as two t127 isolates showed the presence of *sec* and *seh* genes. The strains bearing the *seh* gene are considered a food hazard as this toxin has been unambiguously demonstrated as being capable of potent emetic activity (Le Loir et al. 2003).

Similar to previous studies, we did not identify animal strains of *S. aureus* harboring genes for toxic shock syndrome toxin (*tst*) and PV leukocidin (Gomez-Sanz et al. 2013). Therefore, such strains seem to be specific for human infections.

## Materials and methods

### Bacterial isolates

Between February 2008 and December 2011, 369 dogs and 33 cats were sampled at ten veterinary practices in four cities in northern Poland. One hundred and seventy-two clinically healthy dogs and twenty-one clinically healthy cats were sampled by using sterile swabs from pharynx, nares and rectum. Samples from 197 infected dogs and 12 cats (with dermatitis, external otitis, conjunctivitis, vaginitis, rhinitis and cystitis) were obtained by swabbing affected sites. Between July 2006 and February 2007, swabs were collected on four farms in northern Poland from 71 pigs with skin lesions. Poultry strains were kindly provided by Prof. Alina Wieliczko from the Department of Epizootiology with Clinic for Birds and Exotic Animals, Wroclaw University of Environmental and Life Sciences (Poland). In 2006–2008, samples from poultry with arthritis and septicemia were collected postmortem on 12 farms. Specimens were subcultured onto Columbia blood agar and incubated at 35 °C for 24 h.

Reference enterotoxigenic *S. aureus* strains (F137 and F913) were kindly provided by Prof. Jacek Bania from the Wroclaw University of Environmental and Life Sciences.

### Identification of staphylococcal isolates

Suspected staphylococcal isolates were identified on the basis of colony characteristics, pigment production, Gram-stained appearance and hemolysis. The results were confirmed by using the API ID 32 Staph-system (bioMeriux, Poland), according to the manufacturer’s instructions. In addition, all strains identified as *S. aureus* were analyzed for the presence of species-specific thermostable nuclease gene (*nuc*SA) as described by Baron et al. (2004).

### Methicillin resistance detection

DNA was isolated as described previously, and methicillin resistance was verified by *mecA* gene amplification (Barski et al. 1996).

### Presence of virulence genes

Multiplex PCRs for the detection of exfoliative toxin genes (*eta*, *etb*) and classical staphylococcal PTSAg genes (*sea, seb sec, sed, see* and *tst*) were conducted as previously described (Mehrotra et al. 2000). The genes *seg, sei, sem, sen* and *seo* were detected according to Jarraud et al. (Jarraud et al. 2002), and the detection of *seh*, s*ej*, *sek*, *sel* and *sep* genes followed the protocol published elsewhere (Bania et al. 2006). The amplification of PVL genes (*lukS/lukF*-*PV)* was performed as described by Lina et al. (Lina et al. 1999).

### Genotyping

The *spa* typing was performed as described previously (Harmsen et al. 2003). Sequencing of PCR product was performed with ABI 377 device (Applied Biosystems, Foster City, California). The obtained short sequence repeats (SSRs) were numbered and processed with the Ridom SpaServer software available at http://spaserver.ridom.de.

### Phage typing

Phage typing was performed using the international set of phages, as described by Blair and Williams (Blair and Williams 1961). Initially, the typing was performed at routine test dilution (RTD). Then, the strains that proved non-typeable at RTD were re-typed at 100 × RTD. The strains were subdivided into phage groups I, II, III and V, type 95, mixed group (strains belonging to different lytic groups) and NT (non-typeable at 100 × RTD). The lytic patterns were determined after 18-h incubation at 30 °C.

## Conclusions

Our analysis revealed that the incidence of some toxin genes in *S. aureus* is host specific. Canine isolates t144 of phage group II harbored exfoliative toxin gene (*eta*), and porcine isolates type t1491 representing phage group III showed enterotoxin A gene (*sea).* The enterotoxin gene cluster (*egc1)* and enterotoxin gene *seh* were found in non-typeable isolates from chicken and in one feline isolate type t5447.
